# Measles Vaccines Designed for Enhanced CD8^+^ T Cell Activation

**DOI:** 10.3390/v12020242

**Published:** 2020-02-21

**Authors:** Elena Busch, Kristina D. Kubon, Johanna K. M. Mayer, Gemma Pidelaserra-Martí, Jessica Albert, Birgit Hoyler, Johannes P. W. Heidbuechel, Kyle B. Stephenson, Brian D. Lichty, Wolfram Osen, Stefan B. Eichmüller, Dirk Jäger, Guy Ungerechts, Christine E. Engeland

**Affiliations:** 1 National Center for Tumor Diseases (NCT), Department of Medical Oncology, University Hospital Heidelberg, 69120 Heidelberg, Germany; elena.busch@med.uni-heidelberg.de (E.B.); jessica.albert@nct-heidelberg.de (J.A.); birgit.hoyler@nct-heidelberg.de (B.H.); j.heidbuechel@dkfz-heidelberg.de (J.P.W.H.); dirk.jaeger@nct-heidelberg.de (D.J.); guy.ungerechts@nct-heidelberg.de (G.U.); 2 Clinical Cooperation Unit Virotherapy, German Cancer Research Center (DKFZ), 69120 Heidelberg, Germany; kristina.kubon@nct-heidelberg.de (K.D.K.); johanna.k.mayer@gmx.de (J.K.M.M.); gemma.pidelaserramarti@nct-heidelberg.de (G.P.-M.); 3 Medical Faculty, Heidelberg University, 69120 Heidelberg, Germany; 4 Research Group Mechanisms of Oncolytic Immunotherapy, Clinical Cooperation Unit Virotherapy, National Center for Tumor Diseases (NCT), German Cancer Research Center (DKFZ), 69120 Heidelberg, Germany; 5 Faculty of Biosciences, Heidelberg University and Helmholtz International Graduate School for Cancer Research, 69120 Heidelberg, Germany; 6 McMaster Immunology Research Centre, Department of Pathology and Molecular Medicine, McMaster University, Hamilton, ON L8S 4K1, Canada; kyle.b.stephenson@gmail.com (K.B.S.); blichty@mac.com (B.D.L.); 7 Research Group GMP & T Cell Therapy, German Cancer Research Center (DKFZ), 69120 Heidelberg, Germany; w.osen@dkfz-heidelberg.de (W.O.); s.eichmueller@dkfz-heidelberg.de (S.B.E.); 8 Clinical Cooperation Unit Applied Tumor-Immunity, German Cancer Research Center (DKFZ), 69120 Heidelberg, Germany; 9 Center for Cancer Therapeutics, Ottawa Hospital Research Institute, Ottawa, ON K1H 8L6, Canada

**Keywords:** measles, vaccination, CD8^+^ T cell activation, oncolytic virus, T cell priming, cancer immunotherapy

## Abstract

Priming and activation of CD8^+^ T cell responses is crucial to achieve anti-viral and anti-tumor immunity. Live attenuated measles vaccine strains have been used successfully for immunization for decades and are currently investigated in trials of oncolytic virotherapy. The available reverse genetics systems allow for insertion of additional genes, including heterologous antigens. Here, we designed recombinant measles vaccine vectors for priming and activation of antigen-specific CD8^+^ T cells. For proof-of-concept, we used cytotoxic T lymphocyte (CTL) lines specific for the melanoma-associated differentiation antigen tyrosinase-related protein-2 (TRP-2), or the model antigen chicken ovalbumin (OVA), respectively. We generated recombinant measles vaccine vectors with TRP-2 and OVA epitope cassette variants for expression of the full-length antigen or the respective immunodominant CD8^+^ epitope, with additional variants mediating secretion or proteasomal degradation of the epitope. We show that these recombinant measles virus vectors mediate varying levels of MHC class I (MHC-I)-restricted epitope presentation, leading to activation of cognate CTLs, as indicated by secretion of interferon-gamma (IFNγ) in vitro. Importantly, the recombinant OVA vaccines also mediate priming of naïve OT-I CD8^+^ T cells by dendritic cells. While all vaccine variants can prime and activate cognate T cells, IFNγ release was enhanced using a secreted epitope variant and a variant with epitope strings targeted to the proteasome. The principles presented in this study will facilitate the design of recombinant vaccines to elicit CD8^+^ responses against pathogens and tumor antigens.

## 1. Introduction

The live-attenuated measles vaccines are highly effective, providing at least 95% protection from clinical measles [1]. These vaccines are safe and elicit both B and T cell responses [2]. Reverse genetics systems are available, allowing for insertion of foreign genes into the measles virus genome to generate recombinant vaccines. Following this approach, measles virus has been used as a vector platform to generate vaccines against multiple pathogens, including Dengue virus, HIV, hepatitis B virus, SARS and MERS coronaviruses as well as malaria parasites [3,4,5,6,7,8,9]. A phase I clinical trial with a measles vaccine encoding HIV env has been completed (clinicaltrials.gov NCT01320176). A Schwarz vaccine strain expressing Chikungunya virus (CHIKV) structural proteins has successfully completed phase II. Safety and immunogenicity were demonstrated, with up to 95.9% of patients achieving seroconversion [10]. This vaccine mediates expression of CHIKV virus-like particles. Using a similar approach, a measles-based Lassa virus vaccine has been developed and was shown to protect cynomolgus monkeys from a Lassa virus challenge [11].

The measles vaccine platform has also been used for tumor vaccination. Measles vaccines are oncolytic, i.e., they preferentially replicate in malignant cells compared to normal cells, eventually resulting in selective tumor cell lysis. By releasing tumor antigens in concert with DAMPs (danger-associated molecular patterns) and PAMPs (pathogen-associated molecular patterns) during viral infection, oncolytic virotherapy acts as an antigen-agnostic in situ tumor vaccine [12]. While the herpes virus talimogene laherparepvec has been approved by the FDA and EMA, several other oncolytic viruses are currently in clinical trials, including vaccinia, adeno-, rhabdo- and reoviruses [13]. Oncolytic measles vaccines are under clinical investigation for treatment of several tumor entities, and early trials have indicated the safety and efficacy of this approach [12]. The advantages of oncolytic measles vaccines include the well documented safety record, genomic stability, and the versatility of the reverse genetics system [2,14,15]. Insertion of immunomodulatory genes encoding cytokines, chemokines or immune checkpoint antibodies into the oncolytic vector can enhance anti-tumor immunity [16,17,18,19,20,21,22,23].

As another approach to direct the immune response against the tumor, oncolytic viruses encoding tumor antigens have been developed [24,25,26,27,28,29,30,31]. Different antigen expression strategies have been realized in recombinant viral vectors for tumor vaccination. Kottke et al. and Pulido et al. studied oncolytic vesicular stomatitis virus (VSV) with cDNA libraries from melanoma or prostate cancer [24,25]. Several studies have investigated oncolytic Maraba viruses encoding full-length differentiation or cancer testis antigens [26,27,28,29]. For the treatment of HPV-associated cancers, Atherton et al. generated vectors encoding full-length HPV 16 and 18 E6 and E7 separated by linker sequences to mediate proteasomal degradation [30]. Mühlebach and colleagues have designed recombinant measles vaccines encoding the full-length Claudin 6 tumor antigen either alone or in combination with murine leukemia virus (MLV) gag [31]. The latter strategy enables incorporation of the antigen into MLV virus-like particles (VLPs) to increase immunogenicity. Retroviruses have also been employed successfully for ectopic expression of antigenic T cell epitopes [32].

CD8^+^ cytotoxic T lymphocyte (CTL) responses are essential for efficient anti-viral and anti-tumor immunity. Therefore, induction of robust CTL responses is integral to successful vaccination against viral infections and malignant diseases. In this study, we have developed recombinant measles Schwarz vaccine vectors for CD8^+^ T cell activation. We have designed epitope cassette variants for increased MHC-I epitope presentation and tested their ability to activate CTLs. Using ovalbumin (OVA) and tyrosinase-related protein-2 (TRP-2, synonym L-dopachrome tautomerase) as model antigens, we demonstrate activation of cognate CTLs by recombinant measles vaccine vectors.

## 2. Materials and Methods

### 2.1. Cell Lines and Cell Culture

Vero cells (African green monkey kidney epithelial cells, ATCC CCL-81) were obtained from the American Type Culture Collection (ATCC, Manassas, VA, USA). MC38 cells were a gift from R. Cattaneo (Mayo Clinic, Rochester, MN, USA). B16-OVA cells and the immortalized mouse (C57BL/6) dendritic cell line DC2.4 were obtained from H. Weyd (German Cancer Research Center, Heidelberg, Germany). MC38-hCD46 and B16-OVA-hCD46 cells were generated by lentiviral transduction as described previously [21]. The ovalbumin-specific cytotoxic T cell (CTL) line, which recognizes the H-2K^b^-restricted epitope aa257–264 (SIINFEKL) [33] and the TRP-2-specific CTL line, which recognizes the epitope aa180–188 (SVYDFFVWL) [34]**,** have been described previously [32]. OT-I mice (C57BL/6-Tg(TcraTcrb)1100Mjb/Crl) were bred in the Center for Preclinical Research of the German Cancer Research Center according to institutional guidelines and the German Animal Protection Law. To obtain OT-I T cells, spleens were harvested from untreated OT-I mice. Single-cell suspensions were prepared using a 100 µm mesh and erythrocytes were lyzed using ACK buffer (Lonza, Basel, Switzerland). T cells were isolated using Pan T Cell Isolation Kit II, mouse (Miltenyi Biotech, Bergisch Gladbach, Germany), according to the manufacturer’s protocol.

Vero and MC38-hCD46 cells were cultivated in DMEM + 10% FCS. B16-OVA-hCD46 cells were cultivated in RPMI + 10% FCS. DC2.4 cells were cultivated in RPMI + 10% FCS + 1% HEPES. CTL lines were maintained in α-MEM (Sigma, Taufkirchen, Germany) supplemented with 10% FCS, 4 mM L-glutamine, 100 U/mL penicillin, 100 μg/mL streptomycin and 10 μM β-mercaptoethanol, and restimulated as described previously [35]. In brief, 5% (v/v) supernatant of concanavalin A stimulated (5 μg/mL) rat spleen cell cultures and 25 mM methyl-α-mannopyroside (Sigma) were added to the medium from the second restimulation onwards. Irradiated (33 Gy) syngeneic naïve splenocytes as feeder cells, as well as irradiated (200 Gy) E.G7 (EL4 cells expressing chicken ovalbumin) or RMA/TRP-2 (expressing human tyrosinase-related protein-2), respectively, were used for restimulation.

### 2.2. Cloning, Rescue and Propagation of Recombinant Measles Vaccine Viruses

Recombinant measles viruses (MeV) of the Schwarz vaccine strain (MeVac) were generated using the reverse genetics system originally described by Radecke et al. [36] with the modifications described previously [22,37]. MeVac encoding enhanced green fluorescent protein (eGFP) has been described previously [20]. To obtain cDNA of the ovalbumin and mTRP-2 open reading frames (ORFs), total RNA was extracted from B16-OVA cells using the RNeasy Mini Kit (Qiagen, Hilden, Germany) and cDNA synthesis was performed using the Maxima H Minus First Strand cDNA Synthesis Kit (ThermoFisher, Dreieich, Germany). ORFs were amplified by PCR using the following primers including restriction sites (underlined), a Kozak sequence (forward primers), start or stop codons (bold print), as well as a second stop codon in the case of ovalbumin to comply with the rule of six [38].

ggOVA MluI for (5′→3′) tttacgcgtgccacc**atg**ggctccatcggcg
ggOVA AscI rev (5′→3′) tttggcgcgc**c****tatta**aggggaaacacatctgcca
mTRP-2 MluI for (5′→3′) tttacgcgtgccacc**atg**ggccttgtggga
mTRP-2 AscI rev (5′→3′) tttggcgcgc**c****ta**ggcttcctccgtgt

Ovalbumin and TRP-2 ORFs were inserted into a recombinant MeV harboring an additional transcription unit downstream of the MeV *H* gene [20], yielding pcMeVac OVA and pcMeVac TRP-2. To generate MeV encoding epitope cassette variants (MeVac OVA, MeVac TRP-2), synthetic oligonucleotides were designed and obtained from Eurofins (Ebersberg, Germany). Oligonucleotides included flanking MluI and PauI restriction sites and a Kozak sequence, as well as start and stop codons, and were designed to comply with the rule of six. Oligonucleotides were cloned into MeV harboring an additional transcription unit upstream of the MeV *N* gene [20] to generate pcMeVac SIINFEKL, pcMeVac SVYDFFVWL, pcMeVac Igκ SIINFEKL, and pcMeVac Igκ SVYDFFVWL, pcMeVac Ub-AAY-[SIINFEKL-AAY]_1_, and pcMeVac Ub-AAY-[SIINFEKL-AAY]_2_. To generate pcMeVac Ub-AAY-[SIINFEKL-AAY]_6_ and pcMeVac Ub-AAY-[SVYDFFVWL-AAY]_5_, the sequence encoding the peptide string was obtained by gene synthesis (Eurofins) and cloned into the XbaI and SalI sites of the pN1 NUb AAY plasmid. Subsequently, PCR was performed with primers

pN1 NUb fw (5′→3′) tttacgcgtgccaccatgcagatttttgtgaag
pN1 NUb TRP-2 rev (5′→3′) ttttttgcgcgctcattagtcgacataggctgccaa
pN1 NUb OVA rev (5′→3′) ttttttgcgcgctcattagtcgacataggctgccaa

Sequences of oligonucleotides are presented in Supplementary Table S1.

The rescue and propagation of recombinant viruses were performed as described previously [39]. In brief, Vero cells were seeded in 6-well plates in DMEM + 2% FCS + 50 µg/mL Kanamycin and transfected with 5 µg of the respective pcMeVac anti-genomic plasmid, 500 ng pCDIMER N, 500 ng pDIMER L, and 100 ng pCDIMER P using FuGENE HD Transfection Reagent (Promega, Madison, WI, USA). When syncytia had formed, cells were harvested by scraping and further propagated on Vero cells. Further virus passages were performed at a multiplicity of infection (MOI) of 0.03. Titers were determined by serial dilution titration assay and calculated as cell infectious units per milliliter (ciu/mL) [39].

### 2.3. Growth Curves

To characterize viral replication kinetics, cells were seeded in 12-well plates (1 × 10^5^ cells per well) and infected with designated viruses at MOI 3 in triplicates. To generate one-step growth curves, cells were scraped in media, triplicate samples were pooled, frozen in liquid nitrogen and titrated by serial dilution titration assays.

### 2.4. Western Blot

Cells were seeded in 6-well plates and infected at MOI 3. After 48 h, cell lysates were prepared in RIPA buffer. Protein concentrations were determined by BCA assay (Novagen, Madison, WI, USA) and equal amounts of protein were loaded for SDS-PAGE. Immunodetection of TRP-2 was performed with rabbit polyclonal DCT Antibody (N-terminus; Abcepta, San Diego, CA, USA) at a dilution of 1:1000, and secondary goat anti-rabbit IgG-HRP conjugate (Bethyl, Montgomery, TX, USA) at a dilution of 1:2000. Melanosomes purified from MNT-1 melanoma cells by ultracentrifugation served as positive control. Monoclonal mouse anti-ovalbumin 3G2E1D9 (Santa Cruz Biotechnology, Dallas, TX, USA) at a dilution of 1:1000 and secondary rabbit anti-mouse IgG-HRP conjugate (Bethyl) at a dilution of 1:2000 were used for immunodetection of ovalbumin. Chicken egg white served as positive control. Anti-β-actin-POD clone AC-15 (Sigma) at a dilution of 1:20,000 was used for loading controls.

### 2.5. Synthetic Peptides

OVA aa257–264 (SIINFEKL) and TRP-2 aa180–188 (SVYDFFVWL) peptides were synthesized by Fmoc chemistry [40,41] in a fully automated multiple synthesizer Syro II (MultiSyn Tech, Witten, Germany). The synthesis was carried out on preloaded Wang-Resins. As coupling agent 2-(1H-Benzotriazole-1-yl)-1,1,3,3-tetramethyluronium hexafluorophosphate (HBTU) was used. The material was purified by preparative HPLC on a Kromasil 100–10C 10 µm 120A reverse phase column (20 × 150 mm) using an eluent of 0.1% trifluoroacetic acid in water (A) and 80% acetonitrile in water (B). The peptide was eluted with a successive linear gradient of 25% B to 80% B in 30 min at a flow rate of 10 mL/min. The fractions corresponding to the purified protein were lyophilized. The purified material was characterized with analytical HPLC and MS (Thermo Finnigan LCQ, Thermo Fisher Scientific, Waltham, MA, USA).

### 2.6. Enzyme-Linked Immunospot (ELISpot) Assay

IFNγ ELISpot assays were performed as described previously [35]. Multiscreen HA plates (Millipore, Burlington, MA) were coated with 100 µL capture antibody anti-mouse IFNγ clone R4-6A2 (BD Pharmingen, Heidelberg, Germany) overnight at 4 °C. MC38-hCD46 cells were seeded in 6-well plates (2 × 10^5^ cells per well) and infected at MOI 3. Twenty-four hours after infection, cells were trypsinized and used as target cells (5 × 10^4^ cells per well) for CTLs. After blocking the plates, serial two-fold dilutions of CTLs beginning with 5 × 10^3^ cells per well were performed. CTLs were co-cultured in triplicates with target cells, or with positive controls consisting of synthetic peptides (10 ng per well), uninfected B16-OVA-hCD46 cells (5 × 10^4^ cells per well) or concanavalin A (Sigma, 2 µg per well). After 18 h, cells were removed and the secondary antibody (biotinylated anti-mouse IFNγ antibody (0.2 μg per well, clone XMG1.2; BD Pharmingen)) was added for 2 h at 4 °C, followed by incubation with a streptavidine alkaline phosphatase conjugate (BD Pharmingen, diluted 1:5000 in PBS) at room temperature. BCIP/NBT substrate (Sigma-Aldrich) was added, and the reaction was stopped with water. Spots were counted using a CTL ELISpot reader (CTL Europe, Bonn, Germany). For IFNγ ELISpot assays with DCs, DC2.4 cells were inoculated with MeVac variants at MOI 3. After 24 h, DCs were trypsinized and co-cultures were established with either CTLs or OT-I cells. In each well, 5 × 10^3^ CTLs or isolated OT-I T cells were co-cultured with 5 × 10^4^ DC2.4 cells inoculated with MeVac variants. IFNγ ELISpot assays were performed as described above. For UV inactivation of viruses, virus suspensions (4.1 × 10^6^ ciu in 2 mL OptiMEM) were irradiated with 0.5 J/cm^2^ UV-C light on a UV Stratalinker 2400 (Stratagene, La Jolla, CA, USA). Virus inactivation was confirmed by titration assays.

### 2.7. Flow Cytometry

MC38-hCD46 cells (2 × 10^5^ cells per 6-well plate) or DC2.4 cells (5 × 10^6^ cells per 15 cm plate) were infected at MOI 3 with indicated MeVac variants. For detection of the OVA epitope presented by H-2K^b^, PE anti-mouse H-2K^b^ bound to SIINFEKL Antibody clone 25-D1.16 (BioLegend, San Diego, CA, USA) specific for the SIINFEKL-H-2K^b^ complex was used (0.125 μg per sample). PE Mouse IgG1κ Isotype Control (Biolegend) served as isotype control. For detection of infected cells, anti-measles virus F monoclonal antibody Y503, specific for the measles virus fusion protein, was obtained from D. Gerlier (INSERM, U758, Ecole Normale Supérieure de Lyon, Lyon, France) and used at a 1:40 dilution as described [42]**,** with Mouse F(ab)2 IgG (H+L) APC conjugated (R&D Systems, Minneapolis, MN, USA) at a dilution of 1:50 as second step reagent. Samples were acquired on an LSR II Flow Cytometer or a FACSCanto II Flow Cytometer (BD Biosciences, Heidelberg, Germany) and data were analyzed with FlowJo software (Version X10.0.7r2, Tree Star Inc., Ashland, OR, USA).

To test external loading of MC38-hCD46 cells, cell culture supernatants were passed through a 0.22 µm low protein binding filter (Merck, Darmstadt, Germany). To test external loading of DC2.4 cells, MC38-hCD46 cells (2.5 × 10^5^ cells per well in a 6-well plate) were infected at MOI 3 with indicated MeVac variants. Tumor cells and their culture supernatants were harvested 24 h post-infection, subjected to one freeze-thaw cycle with liquid nitrogen and transferred onto DC2.4 cells (2.5 × 10^5^ cells per well in a 6-well plate). After incubation for 1 h at 37 °C, flow cytometry was performed as described above.

### 2.8. Data Analyses

Graph Pad Prism software (version 6.01; GraphPad Software, LaJolla, CA, USA) was used for data analyses and visualization.

## 3. Results

**Figure 1:**
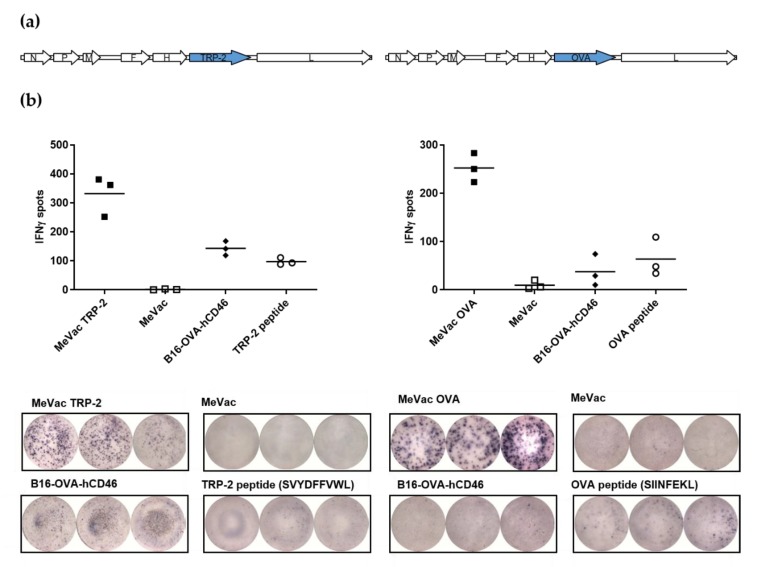
Measles vaccine viruses encoding TRP-2 or ovalbumin (OVA) activate antigen-specific T cells. (**a**) Genome schematics of measles Schwarz vaccine strain viruses encoding TRP-2 (MeVac TRP-2, left panel) and ovalbumin (MeVac OVA, right panel). (**b**) IFNγ ELISpot assay. MC38-hCD46 cells were infected at MOI 3 with MeVac encoding the TRP-2 or OVA antigen or with unmodified MeVac and used as target cells for cytotoxic T lymphocytes (CTLs) which recognize the respective antigen-derived CTL epitope. Synthetic peptides and B16-OVA-hCD46 cells which express both antigens were used as controls. Representative results from one of three independent experiments are shown (upper panels: spot counts, lower panels: magnification of 96-well ELISpot plate). N, P, M, F, H, and L: Measles virus nucleocapsid, phosphoprotein, matrix, fusion, hemagglutinin, and large (polymerase) genes.

To generate measles vaccine vectors for CD8^+^ T cell activation, we initially encoded the full-length murine melanoma-associated differentiation antigen TRP-2 and the model antigen ovalbumin within measles Schwarz vaccine vectors to obtain MeVac TRP-2 and MeVac OVA, respectively (Figure 1a). Due to the size of the antigens (1.6 kb and 1.1 kb, respectively) we chose an additional transcription unit downstream of the MeVac *H* gene for antigen insertion. Cells infected with antigen-encoding MeVac expressed the respective antigen (Supplementary Figure S1). Replication kinetics of MeVac OVA and MeVac TRP-2 in Vero cells were similar to the unmodified measles Schwarz vaccine strain not encoding an additional transgene (Supplementary Figure S2). To test whether MeVac OVA and MeVac TRP-2 can activate cognate T cells, we co-cultured infected target cells with CTL lines, recognizing the H-2K^b^-restricted epitopes OVA aa257–264 (SIINFEKL) or TRP-2 aa180–188 (SVYDFFVWL), respectively. As target cells, we employed MC38-hCD46 cells (MC38 cells transduced to express the MeVac receptor CD46), which are infected by measles vaccine strains (Supplementary Figure S3). ELISpot assays revealed release of IFNγ in co-cultures with MC38-hCD46 cells infected with MeVac encoding the respective antigen, but not unmodified MeVac (Figure 1b).

**Figure 2:**
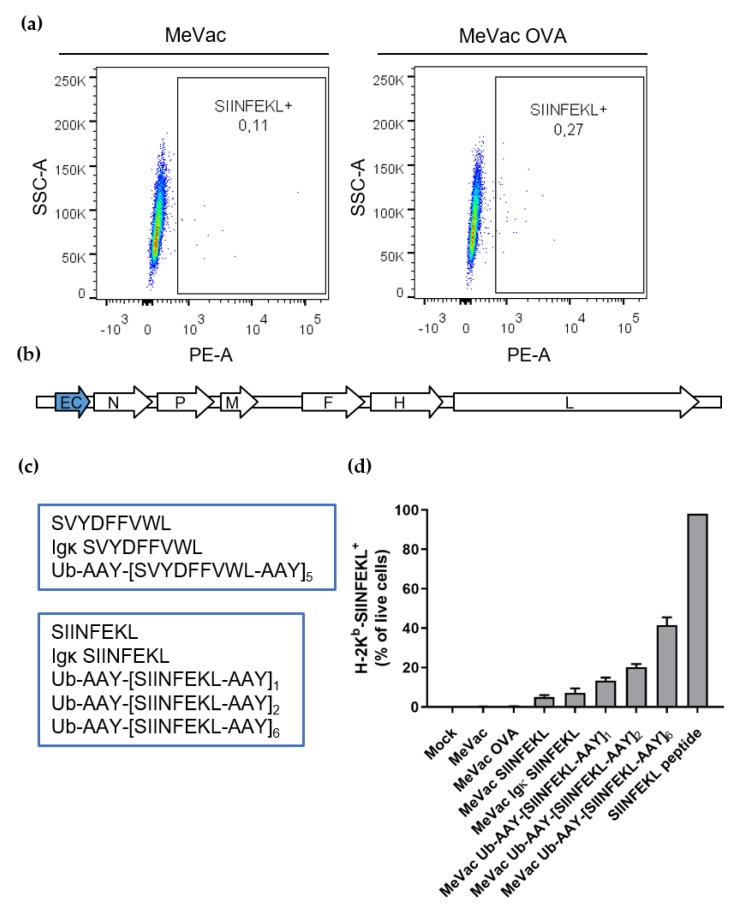
Measles vaccine viruses with epitope cassette variants mediate MHC-I restricted epitope presentation. (**a**) MC38-hCD46 cells were infected with MeVac OVA or unmodified MeVac at MOI 3. Twenty-four hours after infection, flow cytometry was performed with an antibody specific for OVA aa257–264 (SIINFEKL) presented by H-2K^b^. (**b**) Genome schematic of measles Schwarz vaccine strain viruses with epitope cassette (EC) variants. (**c**) Epitope cassette variants for the TPR-2 (top panel) and ovalbumin (bottom panel) CTL epitopes. (**d**) MC38-hCD46 cells were infected with MeVac encoding epitope cassette variants or unmodified MeVac at MOI 3 in triplicates. Twenty-four hours after infection, flow cytometry was performed with an antibody specific for SIINFEKL presented by H-2K^b^. Mean values of triplicates and 95% confidence intervals from one representative of three independent experiments are shown.

Flow cytometry with an antibody specific for SIINFEKL bound to the H-2K^b^ molecule confirmed presentation of the CTL epitope on only 0.27% of infected cells (Figure 2a). Using pre-stimulated CTL lines, this level of presentation was sufficient to elicit strong CTL activation (as shown in Figure 1b). However, in other settings, cell surface expression of epitope-presenting MHC-I molecules may be the limiting factor for an effective CTL response. Therefore, we designed recombinant measles vaccine vectors with different epitope cassette variants to enhance MHC-I-restricted epitope presentation. Instead of encoding the full-length antigen, we inserted variants encoding the immunodominant epitopes only. This enabled insertion into an additional transcription unit upstream of the MeVac *N* gene (Figure 2b). According to the transcription gradient within the measles virus genome, this position mediates higher expression levels [43].

The epitope cassette variants included the epitope only (MeVac SVYDFFVWL, MeVac SIINFEKL), the epitope preceded by the mouse immunoglobulin kappa light chain (Igκ) leader sequence as secretion signal (MeVac Igκ SVYDFFVWL, MeVac Igκ SIINFEKL), as well as epitope strings with an N-terminal ubiquitin and flanked by proteasomal cleavage sites. The latter cassettes were conceptualized based on previous epitope vaccines targeted to the proteasome and on investigations of the epitope flanking residues [44,45,46,47,48,49]. Variants with five (SVDFFVWL) and one, two or six (SIINFEKL) epitope copies were generated (Figure 2c).

MeVac variants were characterized in terms of replication kinetics in Vero and MC38-hCD46 cells (Supplementary Figure S4). A moderate attenuation of MeVac variants compared to unmodified MeVac was observed, especially in MC38-hCD46 cells. At distinct timepoints post infection, MeVac variants with epitope strings showed decreased levels of viral progeny compared to unmodified virus in Vero cells, and MeVac variants encoding full-length OVA or SIINFEKL epitope variants showed attenuation compared to unmodified MeVac in MC38-hCD46 cells. The MeVac fusion protein F was detected on the surface of MC38-hCD46 cells 24 h after infection with each variant (Supplementary Figure S5a). Percentages of MeV F+ cells varied between the constructs, but did not show a clear correlation with replication kinetics. Of note, comparison of MeV F staining with eGFP expression after infection with MeVac eGFP revealed that MeV F staining underestimates the percentage of productively infected cells (Supplementary Figure S5b,c).

Flow cytometry revealed increased H-2K^b^-SIINFEKL expression on cells infected with MeVac encoding epitope variants compared to MeVac OVA (Figure 2d and Supplementary Figure S6). While MeVac SIINFEKL elicited approximately 5% H-2K^b^-SIINFEKL positive cells, MeVac Igκ SIINFEKL elicited approximately 8% positive cells. Variants encoding epitope strings targeted to the proteasome showed increased epitope presentation, which correlated with the number of SIINFEKL repeats. Thus, the MeVac variant encoding a string of six epitopes yielded more than 40% positive cells. Overall, there was no direct correlation between percentages of H-2K^b^-SIINFEKL positive cells and percentages of MeV F+ cells (compare Supplementary Figures S5 and S6), indicating that aside from infectivity, additional factors contribute to the efficacy of epitope presentation of the different MeVac variants.

**Figure 3:**
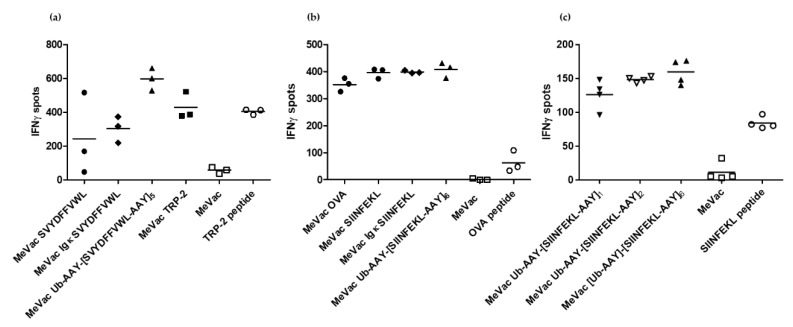
Measles vaccine viruses with epitope cassette variants activate cognate CD8^+^ T cells. MC38-hCD46 cells were infected with indicated MeVac variants at MOI 3 and used as target cells for CTLs which recognize the respective epitope, (**a**): SVYDFFVWL, (**b**) and (**c**): SIINFEKL. After 18 h of co-culture, IFNγ ELISpot assays were performed. Representative results from one of three independent experiments are shown.

IFNγ ELISpot assays confirmed activation of SVYDFFVWL- and SIINFEKL-specific CTLs by cells infected with all epitope cassette variants tested (Figure 3). There were no consistent significant differences in spot numbers between the variants across three independent experiments, again indicating that epitope presentation is not a limiting factor in this setting. However, there was a trend for increased IFNγ release for the TRP-2 epitope string targeted to the proteasome (Figure 3a). In the case of OVA, epitope cassette variants elicited higher mean spot numbers compared to the full length antigen (Figure 3b). Despite slight dose-dependent effects observed for variants with one, two or six epitope copies (Figure 3c), there were no significant differences between the variants containing cassettes with a secretion signal or one or several epitope copies, respectively (Figure 3b,c).

Subsequent experiments focus on the OVA antigen, for which the respective cell lines and reagents were readily available. We tested whether external loading of H-2K^b^ molecules by peptides released from infected cells contributed to the presentation of SIINFEKL. Cell lysates and supernatants from infected cells were collected, cleared by centrifugation and passed through a 0.22 µm low protein binding filter to exclude viral particles. MC38-hCD46 cells treated with lysates and supernatants stained negative for H-2K^b^-SIINFEKL in flow cytometry (data not shown), indicating that in this setting external peptide loading does not contribute to MeVac-mediated epitope presentation and CTL activation.

**Figure 4:**
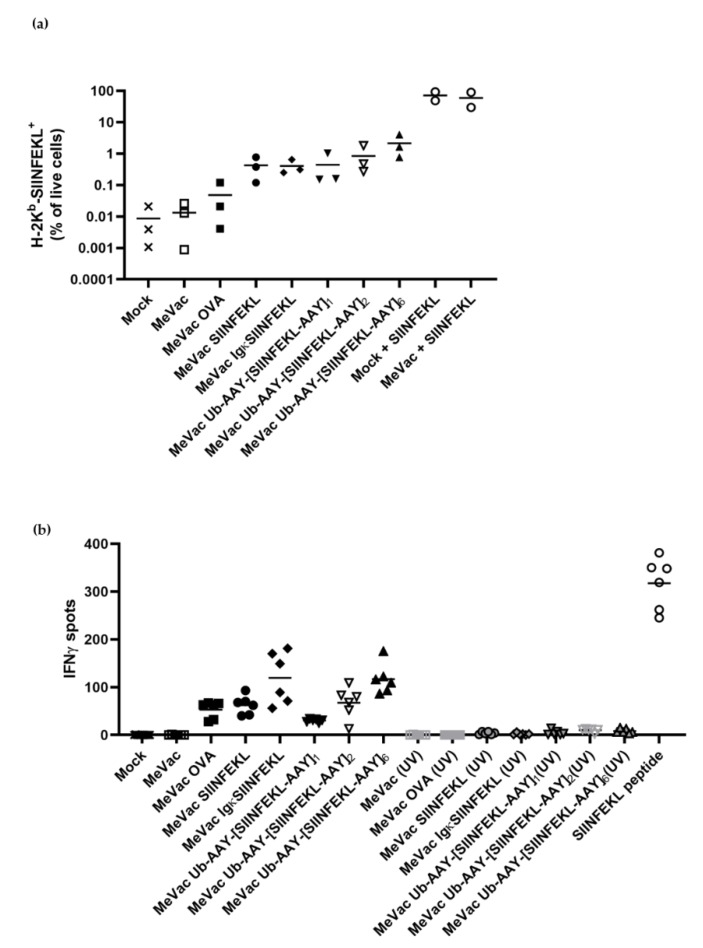
Dendritic cells exposed to measles vaccine viruses with epitope cassette variants mediate epitope presentation by H-2K^b^ and activate cognate T cells. DC2.4 cells were inoculated with recombinant viruses encoding epitope cassette variants at MOI 3. Where indicated, viruses were inactivated by UV irradiation prior to inoculation. (**a**) After 24 h, H-2K^b^-SIINFEKL was detected by flow cytometry with a PE-labeled antibody. Results from three independent experiments are shown with bars indicating mean values for each condition. (**b**) After 24 h, co-cultures with SIINFEKL-specific CTLs were established and IFNγ ELISpot assay was performed. Bars indicate mean values of six replicates for each condition. One representative of three independent experiments with non-inactivated viruses is shown.

Since dendritic cells (DCs) are key to CTL priming and activation, we assessed the MeVac variants in co-cultures of DCs and CTLs. In contrast to MC38-hCD46, the murine DC line DC2.4 shows limited susceptibility to MeVac (Supplementary Figure S7). DC2.4 cells exposed to MeVac encoding SIINFEKL variants showed low percentages of positivity for H-2K^b^-SIINFEKL (Figure 4a). Percentages of H-2K^b^-SIINFEKL positive cells did not directly correlate with MeV F staining, again indicating that infectivity is not the main determinant of epitope presentation. We wondered whether external peptide loading of DCs may contribute to epitope presentation in the context of oncolytic vaccination. To this end, MC38-hCD46 cells were infected with MeVac variants and lyzed 24 h post infection. DCs treated with lysates of infected cells were negative for H-2K^b^-presented SIINFEKL, in contrast to peptide-pulsed DCs (Supplementary Figure S8). Loading of DCs with lysates from MC38-hCD46 cells pulsed with SIINFEKL peptide did not mediate epitope presentation on DCs, either (Supplementary Figure S8, bottom row, left panel). To test whether MeVac-exposed DCs can activate SIINFEKL-specific CTLs, we set up co-cultures and performed IFNγ ELISpot assays. Despite overall low levels of infection and epitope presentation (Supplementary Figure S7, Figure 4a), we observed significant IFNγ secretion in co-cultures with DCs exposed to MeVac encoding full-length ovalbumin, in contrast to unmodified MeVac (Figure 4b). DCs exposed to MeVac encoding epitope cassette variants mediated even stronger CTL activation, as indicated by increased IFNγ secretion (Figure 4b). Variants encoding a secreted epitope or multiple proteasome-targeted epitope copies elicited the highest levels of IFNγ release.

To test whether peptides present in virus suspensions contribute to antigen presentation, we performed flow cytometry for H-2K^b^-SIINFEKL and ELISpot assays with UV-inactivated viruses. UV-inactivated viruses elicited neither SIINFEKL presentation nor IFNγ secretion by SIINFEKL-specific CTLs (Figure 4b), suggesting that de novo peptide expression in productively infected cells is required for CTL activation.

**Figure 5:**
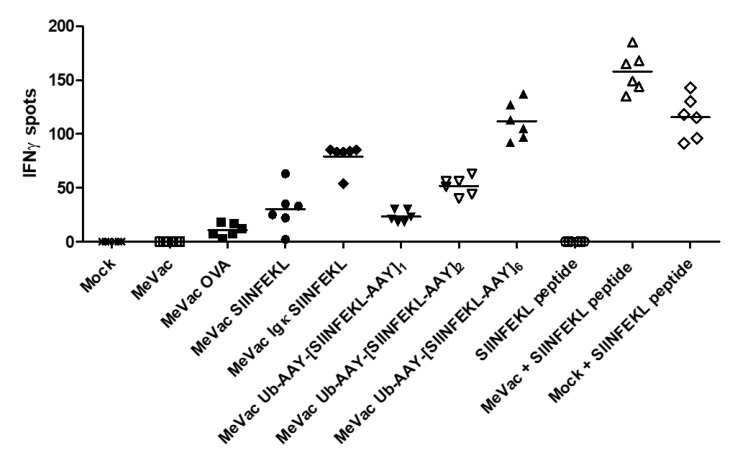
Dendritic cells exposed to measles vaccine viruses with epitope cassette variants can prime cognate T cells. DC2.4 cells were inoculated with measles encoding epitope cassette variants. After 24 h, co-cultures with naïve T cells from OT-I mice were established and IFNγ ELISpot assay was performed. Bars indicate mean value of six replicates for each condition. One representative of three independent experiments is shown.

Priming of naïve T cells is a crucial function of DCs in establishing anti-viral and anti-tumor immunity. To assess T cell priming, we co-cultured DCs exposed to MeVac with naïve OT-I T cells and performed IFNγ ELISpot assays (Figure 5). Despite low levels of infection and H-2K^b^-SIINFEKL positive cells, DCs exposed to MeVac encoding full-length ovalbumin and SIINFEKL epitope cassette variants were able to prime OT-I T cells. MeVac encoding a secreted epitope variant elicited higher levels of IFNγ secretion compared to MeVac encoding SIINFEKL without a secretion signal. There seemed to be a correlation of the number of epitope strings and IFNγ release. While exposure to SIINFEKL peptide alone was insufficient for T cell priming, mock treated DCs pulsed with SIINFEKL peptide induced IFNγ expression by OT-I T cells. Interestingly, DCs exposed to MeVac and subsequently pulsed with SIINFEKL elicited higher levels of IFNγ secretion. These results show that MeVac encoding CD8^+^ T cell epitopes can mediate priming of naïve T cells by professional antigen presenting cells and may promote DC function.

## 4. Discussion

Cytotoxic T cell responses are essential for robust anti-viral and anti-tumor immunity. Strategies to induce CTL responses by vaccination include peptide and DNA vaccines, targeting of DCs and immunization with viral vectors [50]. Herein, we sought to utilize measles vaccine vectors for activation of antigen-specific CTLs.

We encoded either the full-length antigens or epitope cassette variants within the measles Schwarz vaccine backbone. Epitope cassette variants encoded either the CTL epitope only, the epitope preceded by a secretion signal, or epitope strings targeted for proteasomal degradation. Using co-cultures of CTLs and cells treated with measles vaccines, we showed that these vaccines can activate cognate T cells. We used both virus-infected tumor cells and DCs exposed to the virus. Similar epitope variants have been tested previously, for instance in DNA vaccination against HIV [51]. In that study, variants targeted to the endoplasmic reticulum and especially the proteasome induced stronger immune responses.

Studying a measles vaccine encoding the hepatitis B surface antigen (HBsAg), Singh et al. showed that UV-inactivated vaccine did not elicit anti-HBsAg responses and concluded that viral replication is required for effective vaccination [6]. The recombinant measles vaccine variants reported here all replicate efficiently, showing replication kinetics similar to the unmodified vaccine strain. Additionally, in the present study, UV inactivation abrogated replication and T cell activation.

In co-cultures of MeVac-susceptible, infected MC38-hCD46 cells and activated CTL lines, all vaccine variants elicited similar levels of IFNγ release by cognate T cells, despite higher levels of MHC-I–restricted epitope presentation with epitope cassette variants. Due to the transcription gradient within the measles virus genome [43], higher expression levels of antigenic peptides can be expected for epitope cassettes further upstream within the genome. However, the trade-off between transgene size and viral replicative fitness should be considered. In settings where MHC-I–restricted epitope presentation is limiting, epitope cassette variants may be beneficial. Accordingly, in co-cultures of murine DCs, which show limited susceptibility to MeVac, epitope cassette variants mediated more pronounced CTL activation compared to MeVac encoding the full-length antigen. Epitope cassette design, respective antigen processing mechanisms, genomic position of the epitope cassette, and viral replication kinetics may contribute to the observed differences in activation levels.

Rennick et al. have previously studied viral spread after intramuscular vaccination of macaques with the Edmonston-Zagreb measles vaccine strain (MV-EZ), encoding GFP as a reporter. They found that macrophages and DCs are the primary target cells after MV-EZ vaccination, showing productive infection, as indicated by detection of intracellular viral proteins [52]. In the present study, we showed that DCs can present virus-encoded CTL epitopes after exposure to measles vaccines. Importantly, we demonstrated that DCs can also prime epitope-specific naïve CTLs. Since we had tools to study OVA-specific CTL activation in a murine system, we used murine DCs for these experiments. We assume that human DCs may be more effective, as they express the MeVac receptor CD46 [53] and measles virus is adapted to human cells. Moreover, measles vaccines have been shown to promote maturation of human DCs without compromising DC viability [53]. Further, both conventional and plasmacytoid human DCs were shown to cross-present tumor antigens and activate tumor-specific CTLs after exposure to measles virus-infected tumor cells [53,54]. Bolton et al. previously showed that a measles vaccine vector can be used to prime SIV Gag-specific T cells in macaques, which could be amplified by a heterologous adenovirus boost [55]. Taken together, these data indicate that measles vaccines can be utilized for induction and promotion of antigen-specific CD8^+^ T cell responses.

Despite higher levels of MHC-I-restricted epitope presentation and IFNγ release with MeVac encoding epitope cassette variants, encoding full-length antigens offers the possibility to induce responses against multiple epitopes of one antigen, including epitopes recognized by T helper (Th) cells, which contribute to CTL responses [56]. Alternatively, distinct additional Th and CTL epitopes could be inserted into the MeVac vector, which has been shown to have a coding capacity of up to 6 kb [57]. However, when designing multiepitope vaccines, factors governing antigen dominance such as T cell precursor frequency, relative epitope abundance and MHC binding affinity should be taken into account [58,59,60].

T cells require three signals for full activation, including T cell receptor engagement by the MHC-epitope complex and co-stimulation by molecules on the surface of the antigen-presenting cell. As discussed above, measles vaccines can contribute to these two signals by inducing MHC presentation of encoded epitopes and by promoting DC maturation. As a third signal, soluble factors promoting T cell differentiation are required and have been shown to be released in the context of measles vaccination [61]. Furthermore, transgenes providing “signal 3” for CD8^+^ T cell activation, such as pro-inflammatory cytokines [62], could be incorporated into the vector. For instance, measles vaccine vectors encoding IFNβ and interleukin-12 have been developed previously [20,22,63]. Additional transgenes to promote DC function, CTL induction, proliferation, differentiation and to prevent CTL exhaustion can be inserted into the measles vaccine vector. Previous examples include granulocyte-macrophage colony-stimulating factor (GM-CSF), interleukin-15 as well as immune checkpoint blocking antibodies [17,19,22,23].

Measles vaccine strains have been used for oncolytic virotherapy and have been shown to induce anti-tumor CTL responses via tumor vaccination effects [17,20,23]. The present study suggests that measles vaccines can also be designed to promote CTL responses against specific tumor antigens by encoding the antigens or their immunodominant epitopes in the viral vector. Other oncolytic virus platforms, such as VSV and Maraba virus, have been used for this purpose [24,25,26,27,28,30,64]. Though not effective as a priming vector, Maraba viruses encoding tumor antigens have been shown to efficiently boost CTL-mediated anti-tumor immunity by infection of splenic follicular B cells [29,65]. Thus, Maraba virus is currently applied in heterologous prime-boost settings with replication-deficient adenoviral vectors [64]. Previous work by Mühlebach and colleagues [31] and the present data support the notion that measles vaccines can be used for priming in oncolytic vaccination regimens.

For this proof-of-concept study, we carried out experiments in established in vitro model systems. Clearly, in vivo experiments are required to study efficacy as well as the interplay with other components of the immune system and to establish dosing and scheduling regimens. The model commonly used for testing of recombinant measles vaccines are IFNAR^−/−^-CD46Ge mice, which express the measles vaccine strain receptor CD46 but are deficient in the type I interferon receptor [3]. Considering the role of type I interferon in anti-tumor immunity [66], this model seems unsuitable for study of oncolytic vaccines. Future studies should address in vivo effects in more refined models of measles vaccination.

Tumor cells often acquire defects in the MHC-I antigen processing machinery [67]. Therefore, epitope cassette variants which facilitate epitope presentation may prove beneficial in oncolytic vaccination settings. In this regard, the insertion of secretion signals as presented here could represent one viable strategy.

Pre-existing anti-measles immunity due to prior infection or immunization is often considered an obstacle to implementing MeVac-based therapeutics. Strategies to circumvent premature clearance have been developed, including chimeric viruses and alternative Paramyxovirus platforms [68,69,70]. However, pre-existing anti-measles immunity did not affect anti-CHIKV immune responses in human subjects [10]. In the context of oncolytic virotherapy, pre-existing anti-viral immunity may even be conducive to anti-tumor efficacy [21,71,72].

Taken together, this study demonstrates that measles vaccines can be designed for effective priming and activation of specific CD8^+^ T cells. These design principles can be adopted for immunization against viral and tumor antigens to develop T cell vaccines against pathogens and malignant diseases.

## Supplementary Materials

The following are available online at https://www.mdpi.com/1999-4915/12/2/242/s1.
