# Supplementary material for: Measles Vaccines Designed for Enhanced CD8+ T Cell Activation
Source: Viruses. 2020 Feb 21;12(2):242. doi: 10.3390/v12020242 (PMC7077255; doi:10.3390/v12020242)
Supplement: Supplementary file 1 [file viruses-12-00242-s001.pdf]

Supplementary Materials:

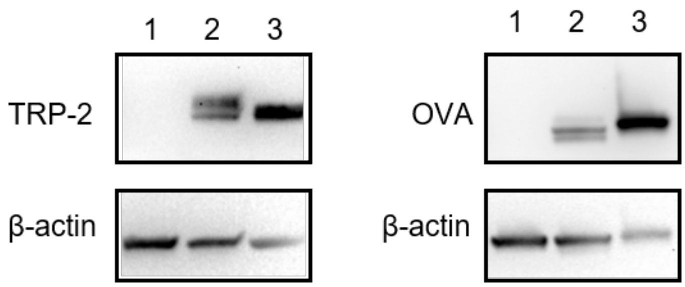

Supplementary Figure 1. Antigen expression after infection with MeVac encoding TRP-2 or OVA. Vero cells were subjected to mock infection or infected with MeVac encoding the TRP-2 or OVA antigen at MOI 3. After 48 h, cells were lyzed and Western blot was performed using antigen-specific antisera. Lane 1: mock; lane 2: MeVac TRP-2, MeVac OVA; lane 3: positive control (TRP-2: melanosomes; OVA: chicken egg white).

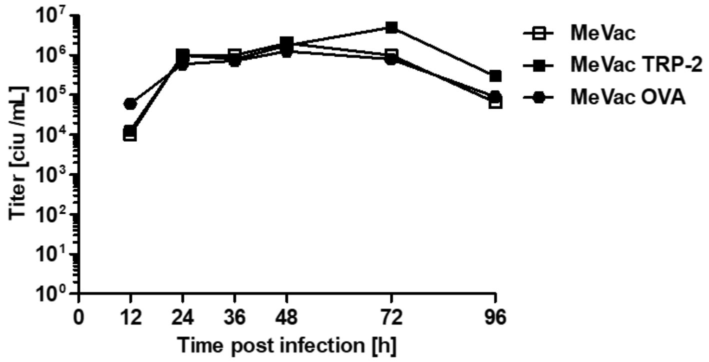

Supplementary Figure 2. Replication kinetics of measles vaccine viruses encoding antigens. To generate one-step growth curves, Vero cells were inoculated with MeVac variants at MOI 3 in triplicates. At designated time points, cells were harvested, triplicate samples were pooled and viral progeny were quantified by titration assays.

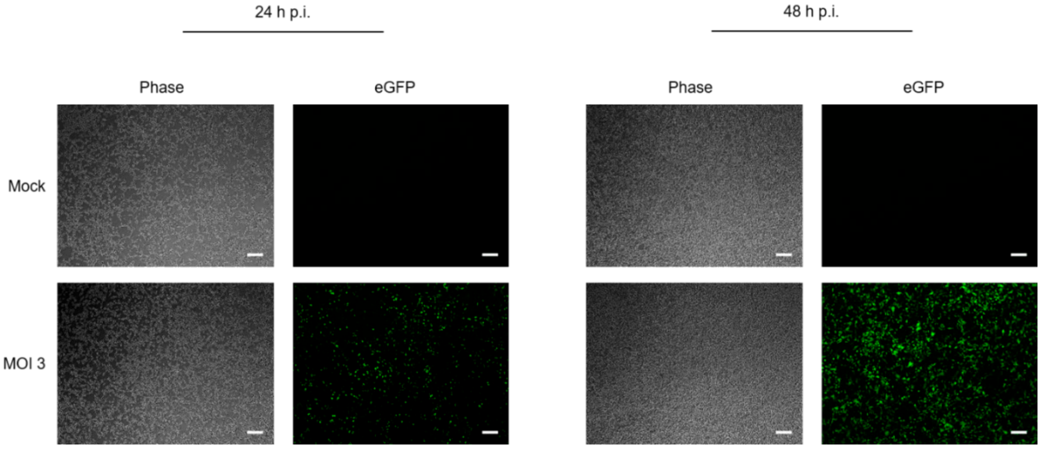

Supplementary Figure 3. The measles Schwarz vaccine strain infects MC38-hCD46 cells. MC38-hCD46 cells were subjected to mock infection or inoculated with MeVac encoding eGFP. Images were acquired 24 and 48 h post infection (p.i.). Scale bars: 200 μm.

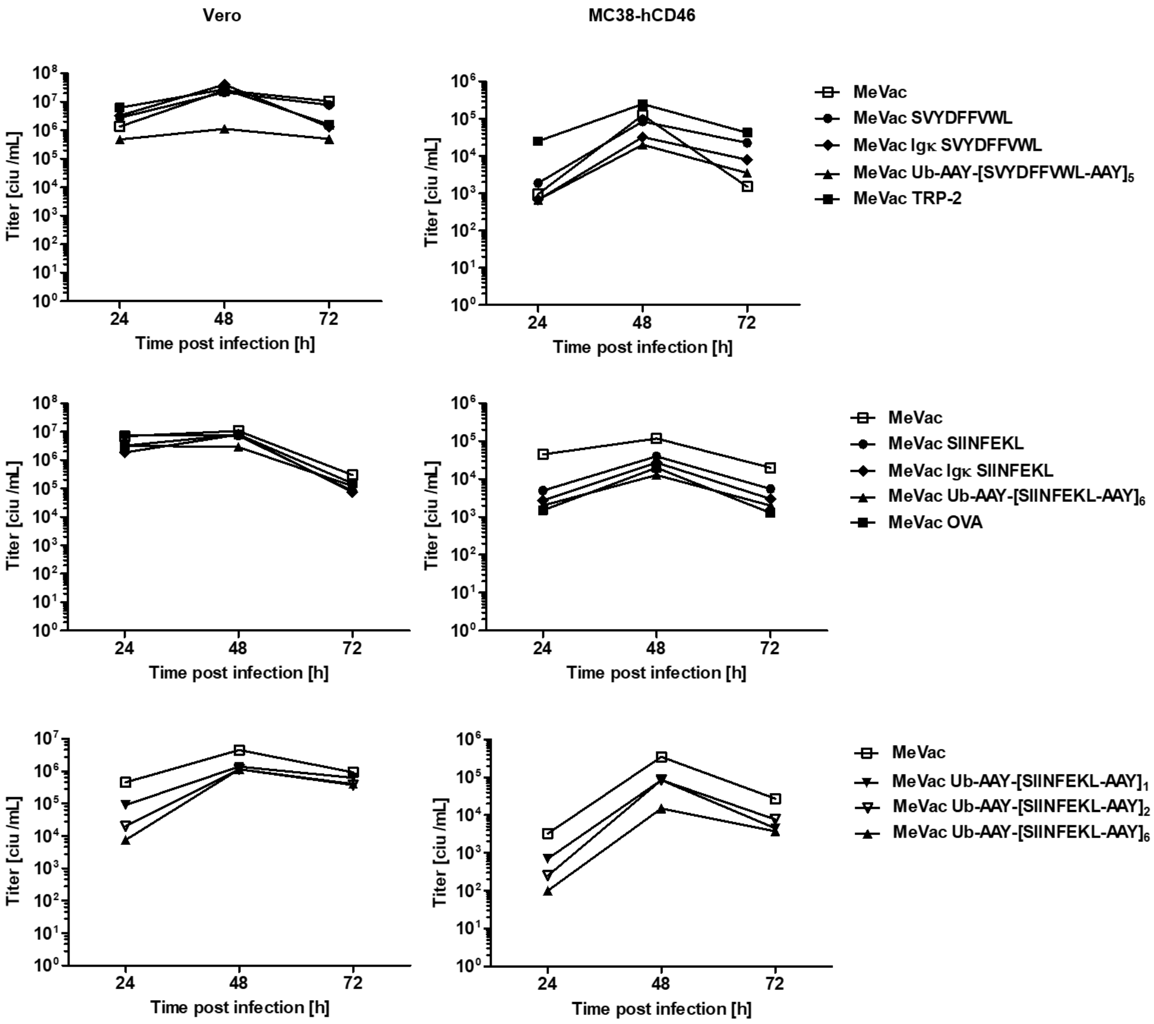

Supplementary Figure 4. Replication kinetics of vectors with epitope cassette variants. Vero (left panels) and MC38-hCD46 cells (right panels) were inoculated with MeVac variants at MOI 3 in triplicates. At designated time points, cells were harvested, triplicates pooled and viral progeny were quantified by titration assays.

(a)

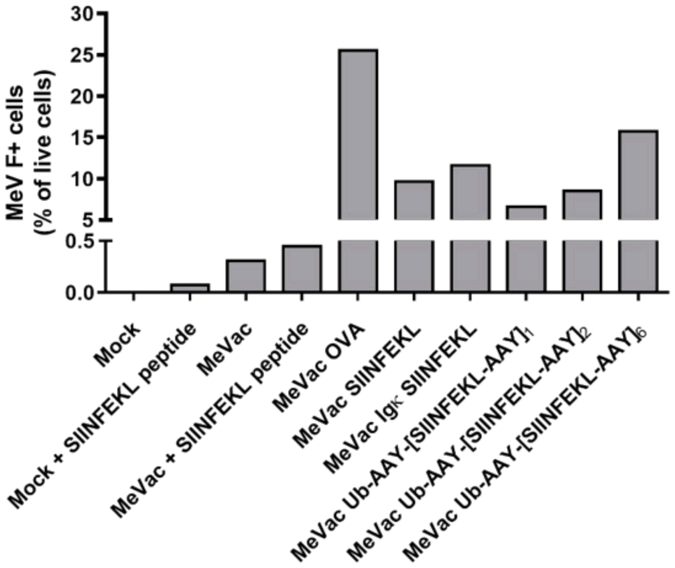

(b)

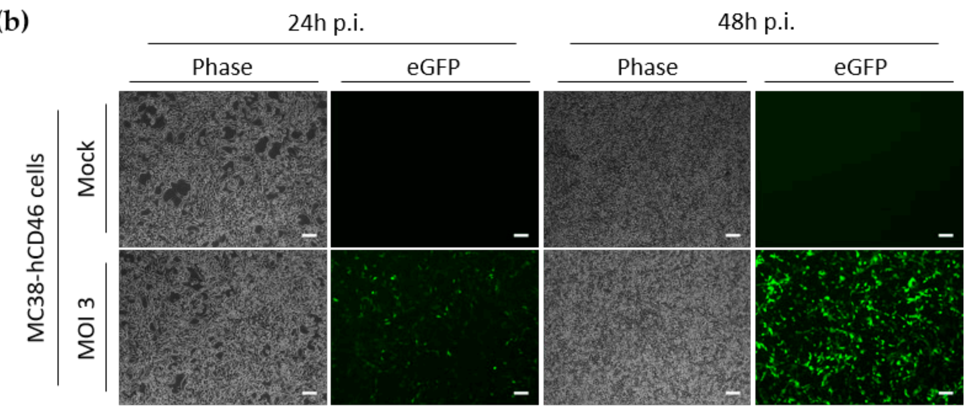

(c)

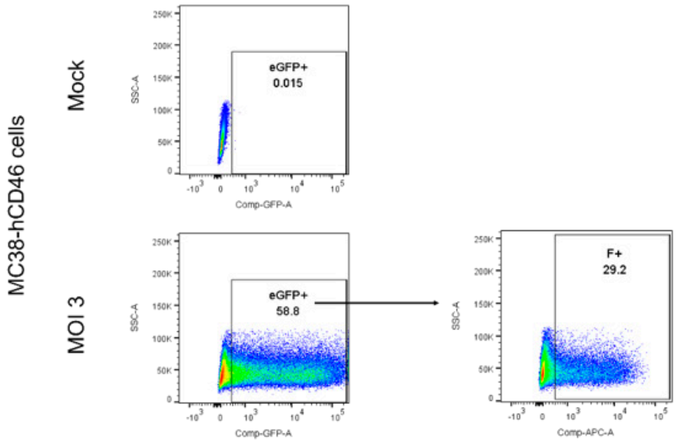

Supplementary Figure 5. MC38-hCD46 show productive infection with recombinant MeVac harboring epitope cassette variants. (a) MC38-hCD46 cells were inoculated with indicated viruses at MOI 3. At 24 h post infection (p.i.), cells were stained with an antibody specific for the MeV fusion protein F. **(b)** MC38-hCD46 cells were infected with MeVac encoding eGFP and images were acquired after 24 and 48 h. Scale bars: 100 μm. **(c)** MeV F staining underestimates MeVac productive infection.

Cells shown in (b) were stained with an MeV F-specific antibody at 24 h post infection. Percentages of F+ of eGFP+ cells are shown.

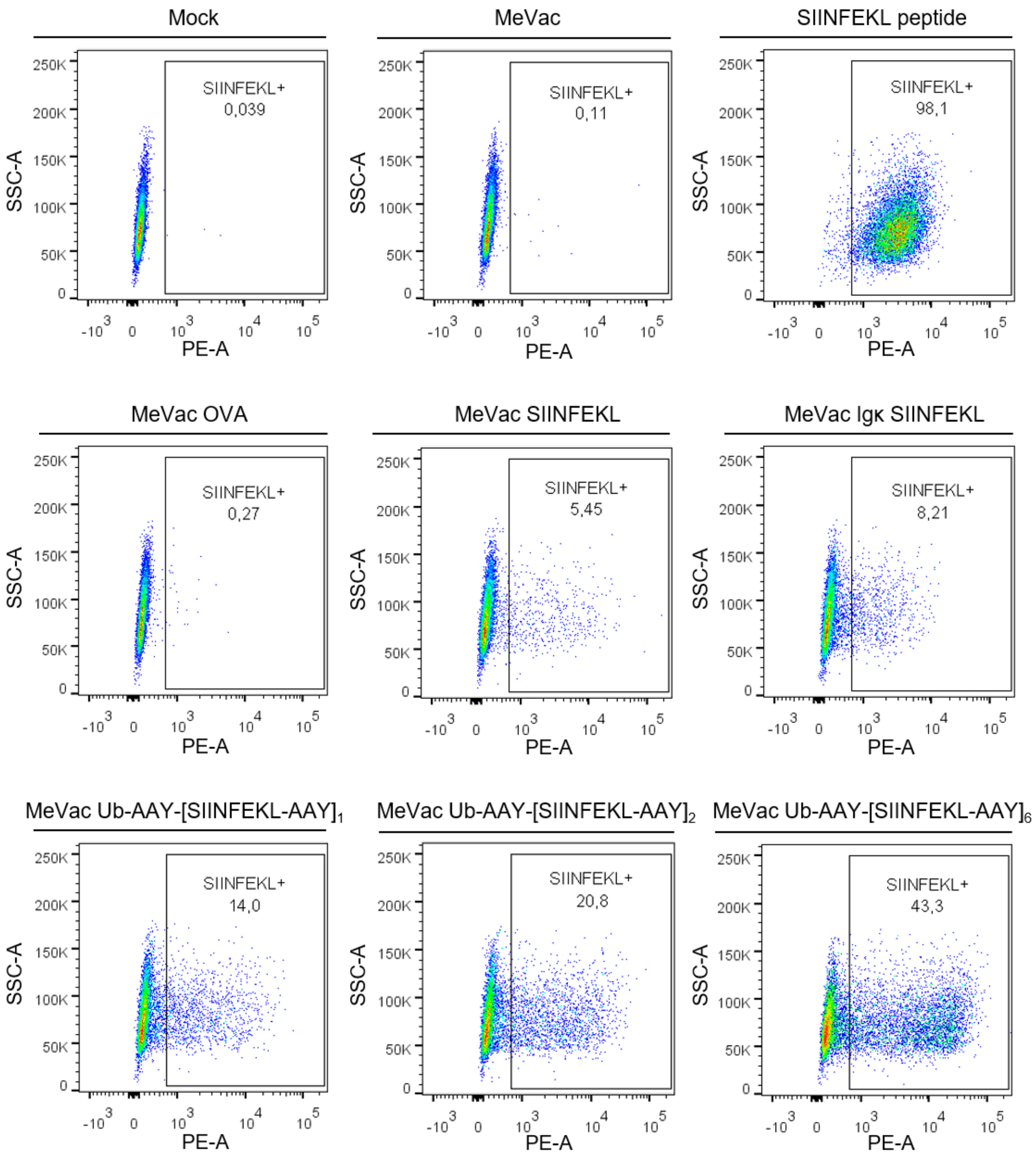

Supplementary Figure 6. Epitope presentation after infection with MeVac variants. MC38-hCD46 cells were infected with indicated MeVac variants at MOI 3. Twenty-four hours after infection, flow cytometry was performed with a PE-conjugated antibody specific for H-2K^b-SIINFEKL. For each variant, one representative sample of three replicates is shown. Cells pulsed with SIINFEKL peptide (upper right panel) served as positive control. Note that the plots for MeVac and MeVac OVA are also displayed in Figure 2a.

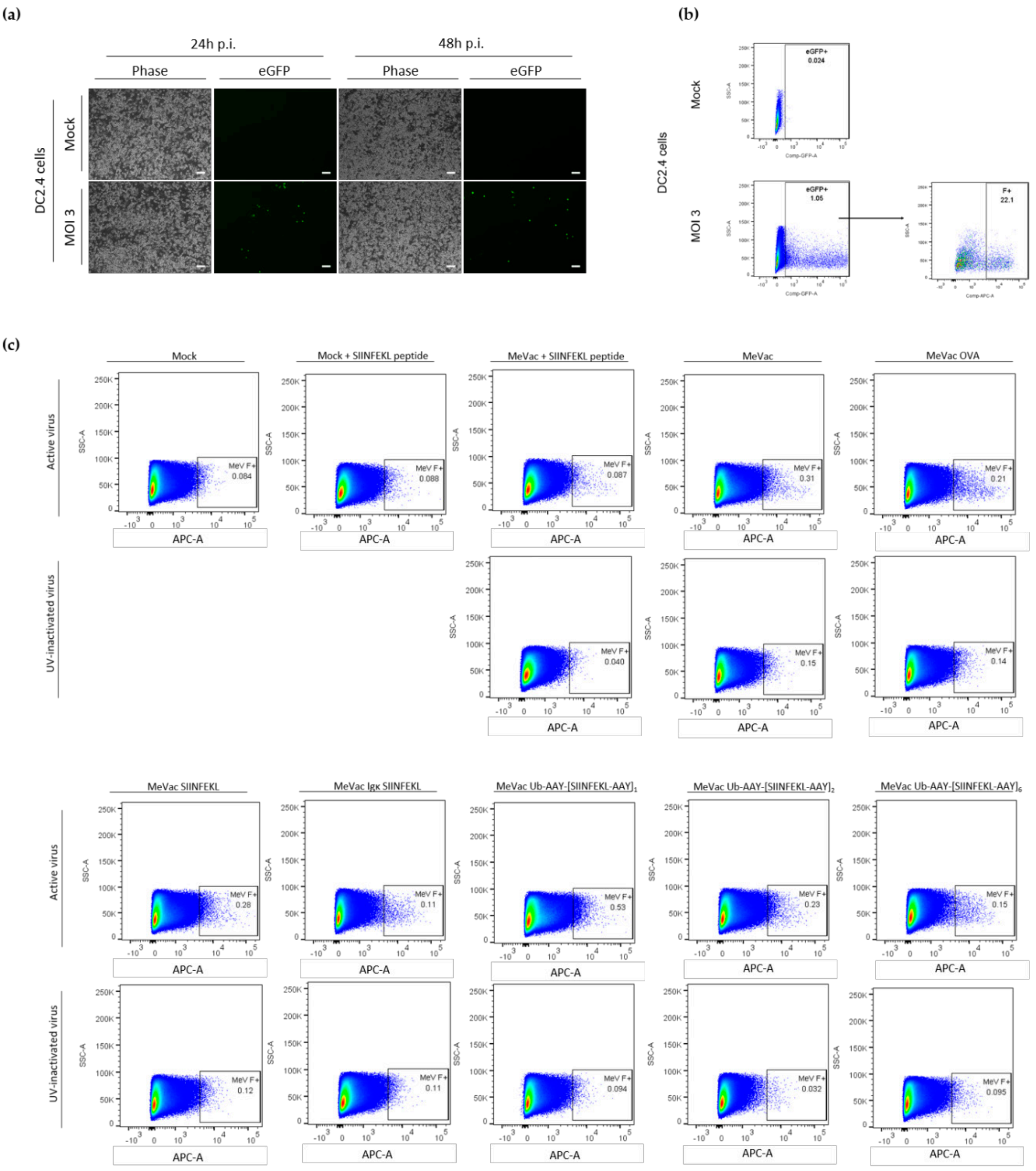

Supplementary Figure 7. Murine dendritic cells show limited susceptibility to measles vaccine virus. (a) DC2.4 cells were inoculated with a measles vaccine variant encoding eGFP at MOI 3. Images were acquired 24 and 48 h post infection. Scale bars: 100 µm. **(b)** At 24 h post infection, flow cytometry was performed. eGFP+ cells and MeV F+ cells of eGFP+ cells are shown. **(c)** DC2.4 cells were inoculated with indicated MeVac variants at MOI 3. After 24 h, flow cytometry was performed with an antibody specific for the MeV fusion protein F. Representative data from three independent experiments are shown.

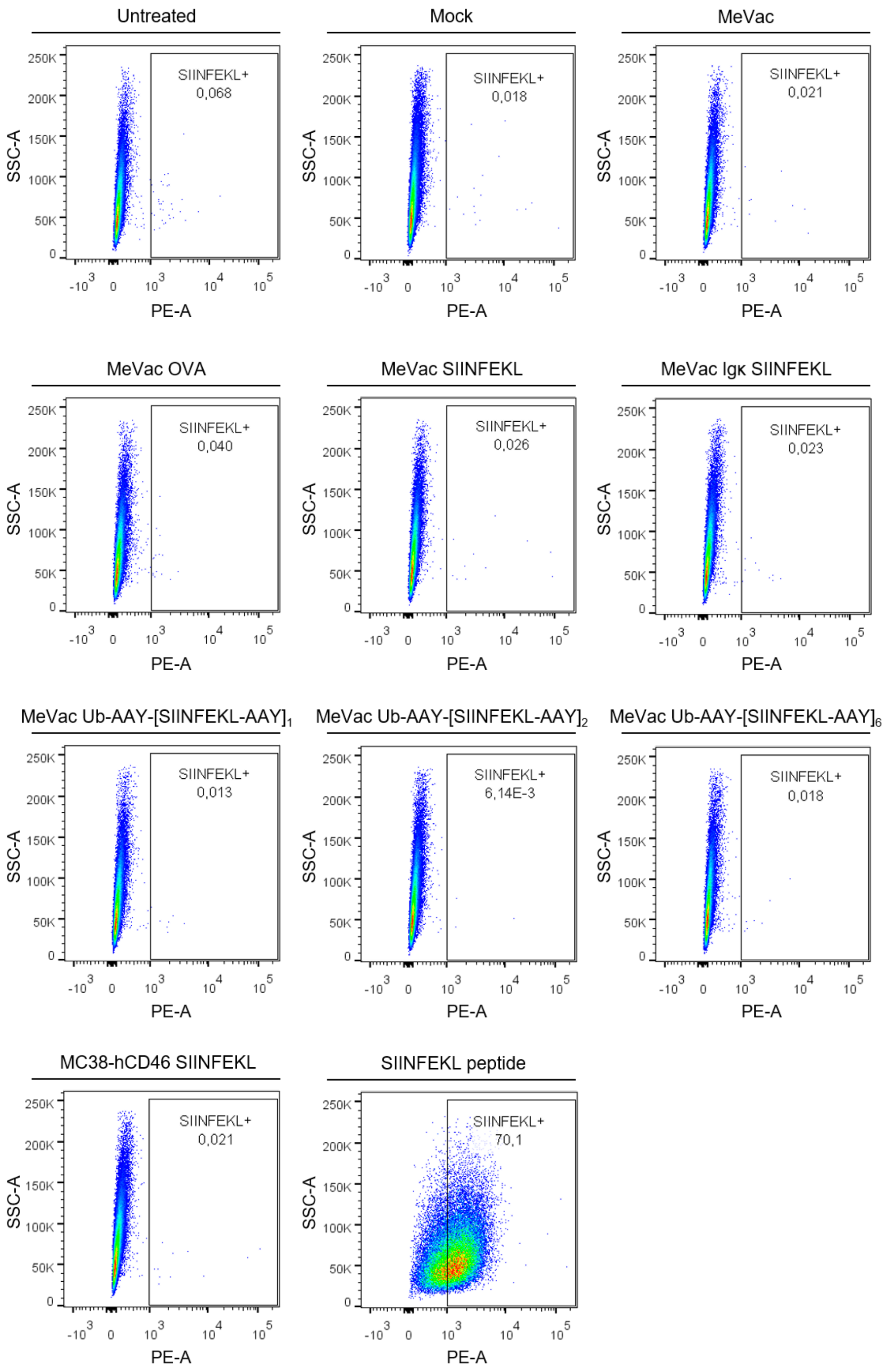

Supplementary Figure 8. External peptide loading does not contribute to epitope presentation by dendritic cells. MC38-hCD46 cells were infected with indicated MeVac variants at MOI 3. After 24 h, cells were lyzed by one freeze-thaw cycle. As control, MC38-hCD46 cells were loaded with SIINFEKL peptide and then lyzed (MC38-hCD46 SIINFEKL). Lysates were cleared by centrifugation. DC2.4 cells were inoculated with lysates or pulsed with SIINFEKL peptide for one hour (bottom row, right panel) and flow cytometry for SIINFEKL bound to H-2K^b was performed with a PE-labeled antibody.

Supplementary Table 1. Oligonucleotide sequences for cloning of epitope cassette variants. [SVYDFFVWL-AAY]$_5$ and [SIINFEKL-AAY]$_6$ were obtained by gene synthesis.

Name	Sequence (5' → 3')
SVYDFFVWL fw	CGCGTGCCACCATGAGCGTGTACGACTTCTTTGTCTGGCTGTAATGAG
SVYDFFVWL rev	CGCGCTCATTACAGCCAGACAAAGAAGTCGTACACGCTCATGGTGGCA
IgK SVYDFFVWL fw	CGCGTGCCACCATGGAGACTGACACCCTGCTTCTGTGGGTGTTGCTGCTCTGGGTTCCT GGATCCACAGGCGATAGCGTCTACGACTTCTTTGTGTGGCTGTGATAAG
IgK SVYDFFVWL rev	CGCGCTTATCACAGCCACACAAAGAAGTCGTAGACGCTATCGCCTGTGGATCCAGGAAC CCAGAGCAGCAACACCCACAGAAGCAGGGTGTCAGTCTCCATGGTGGCA
SIINFEKL fw	CGCGTGCCACCATGAGCATTATCAACTTCGAGAAGCTGTGAG
SIINFEKL rev	CGCGCTCACAGCTTCTCGAAGTTGATAATGCTCATGGTGGCA
IgK SIINFEKL fw	CGCGTGCCACCATGGAAACCGACACTCTGCTCCTGTGGGTCTTGCTGCTTTGGGTGCCT GGATCCACAGGCGATAGCATCATTAACTTCGAGAAGCTGTGAG
IgK SIINFEKL rev	CGCGCTCACAGCTTCTCGAAGTTAATGATGCTATCGCCTGTGGATCCAGGCACCCAAAG CAGCAAGACCCACAGGAGCAGAGTGTCGGTTTCCATGGTGGCA
[SIINFEKL-AAY]$_1$ fw	CTAGATCTATAATCAACTTTGAAAAGCTGGCAGCCTACG
[SIINFEKL-AAY]$_1$ rev	TCGACGTAGGCTGCCAGCTTTTCAAAGTTGATTATAGAT
[SIINFEKL-AAY]$_2$ fw	CTAGATCTATAATCAACTTTGAAAAGCTGGCAGCCTACAGCATTATCAATTTCGAGAAGC TTGCAGCGTACG
[SIINFEKL-AAY]$_2$ rev	TCGACGTACGCTGCAAGCTTCTCGAAATTGATAATGCTGTAGGCTGCCAGCTTTTCAAA GTTGATTATAGAT
[SVYDFFVWL-AAY]$_5$	TCTAGAAGCGTGTATGACTTCTTTGTTTGGCTCGCTGCTTACTCTGTCTACGACTTCTTCG TTTGGCTGGCAGCGTATTCCGTGTATGATTTCTTTGTATGGCTGGCAGCCTACAGTGTGT ACGACTTCTTTGTGTGGCTGGCTGCCTATTCAGTGTACGATTTCTTTGTCTGGCTTGCCG CCTACGTCGAC
[SIINFEKL-AAY]$_6$	TCTAGATCTATAATCAACTTTGAAAAGCTGGCAGCCTACAGCATTATCAATTTCGAGAAG CTTGCAGCGTACTCAATCATCAATTTCGAAAAGCTGGCTGCCTATTCCATTATCAACTTTG AGAAACTGGCTGCCTACAGTATCATAAATTTCGAGAAACTCGCTGCCTATAGCATTATTA ACTTTGAGAAGTTGGCAGCCTATGTCGAC
